# Glycine betaine increases salt tolerance in maize (*Zea mays* L.) by regulating Na^+^ homeostasis

**DOI:** 10.3389/fpls.2022.978304

**Published:** 2022-09-30

**Authors:** Mingyuan Zhu, Qiuxia Li, Yushi Zhang, Mingcai Zhang, Zhaohu Li

**Affiliations:** College of Agronomy and Biotechnology, China Agricultural University, Beijing, China

**Keywords:** glycine betaine, maize, salt tolerance, Na^+^ homeostasis, PM H^+^-ATPase, photosynthesis capacity, antioxidant activity

## Abstract

Improving crop salt tolerance is an adaptive measure to climate change for meeting future food demands. Previous studies have reported that glycine betaine (GB) plays critical roles as an osmolyte in enhancing plant salt resistance. However, the mechanism underlying the GB regulating plant Na^+^ homeostasis during response to salinity is poorly understood. In this study, hydroponically cultured maize with 125 mM NaCl for inducing salinity stress was treated with 100 μM GB. We found that treatment with GB improved the growth of maize plants under non-stressed (NS) and salinity-stressed (SS) conditions. Treatment with GB significantly maintained the properties of chlorophyll fluorescence, including Fv/Fm, ΦPSII, and ΦNPQ, and increased the activity of the antioxidant enzymes for mitigating salt-induced growth inhibition. Moreover, GB decreased the Na^+^/K^+^ ratio primarily by reducing the accumulation of Na^+^ in plants. The results of NMT tests further confirmed that GB increased Na^+^ efflux from roots under SS condition, and fluorescence imaging of cellular Na^+^ suggested that GB reduced the cellular allocation of Na^+^. GB additionally increased Na^+^ efflux in leaf protoplasts under SS condition, and treatment with sodium orthovanadate, a plasma membrane (PM) H^+^-ATPase inhibitor, significantly alleviated the positive effects of GB on Na^+^ efflux under salt stress. GB significantly improved the vacuolar activity of NHX but had no significant effects on the activity of V type H^+^-ATPases. In addition, GB significantly upregulated the expression of the PM H^+^-ATPase genes, *ZmMHA2* and *ZmMHA4*, and the Na^+^/H^+^ antiporter gene, *ZmNHX1.* While, the V type H^+^-ATPases gene, *ZmVP1*, was not significantly regulated by GB. Altogether these results indicate that GB regulates cellular Na^+^ homeostasis by enhancing PM H+-ATPases gene transcription and protein activities to improve maize salt tolerance. This study provided an extended understanding of the functions of GB in plant responses to salinity, which can help the development of supportive measures using GB for obtaining high maize yield in saline conditions.

## Introduction

Salinity affects over 6% of lands worldwide and about one-fifth of the agricultural land area (Munns and Tester, 2008; FAO, 2020). Soil salinization is becoming a major obstacle for agricultural production and crop yield owing to the over-exploitation of underground water and fertilizer overuse (Shabala, 2013; Hassani et al., 2021). The effects of climate change are expected to worsen the situation, especially in arid and semi-arid regions (Hassani et al., 2021). Planting crops in saline soils of coastal regions could be a potential way for grain production (Xie et al., 2017). Therefore, improving the salt tolerance of crop plants is important for achieving food security and reaching sustainable development goals in future agricultural production (Shabala, 2013; Wungrampha et al., 2018).

Soil salinity represses plant growth and development *via* complex processes, which are mainly classified into three categories, namely, osmotic stress, ion toxicity, and oxidative stress (Munns, 2002; Yang and Guo, 2018). Correspondingly, plants have developed several strategies for coping with high salinity in the living environment. In order to adapt to osmotic stress, plants accumulate compatible osmolytes, including proline, glycine betaine (GB), and soluble sugars, which help maintain cellular water potential and membrane stability (Apse and Blumwald, 2002; Szabados and Savouré, 2010). Plants cope with metabolic damages resulted from the high concentration of Na^+^ in cytosol by regulating the membrane ion transporters to help maintain a suitable cellular Na^+^/K^+^ homeostasis (Serrano et al., 1999). Plants employ two important strategies for alleviating cellular Na^+^ cytotoxicity, including the SALT OVERLY SENSITIVE1 (SOS1)-mediated cytosolic exclusion of Na^+^, and the vacuolar sequestration of Na^+^ mediated *via* Na^+^/H^+^ EXCHANGER1 (NHX1) (Wu, 2018). Additionally, the root-to-shoot delivery of Na^+^ and the accumulation of K^+^ in the shoot mediated *via* the genes of the high-affinity K^+^ channel (HKT) family, including *ZmHKT1* and *ZmHKT2*, are also important strategies for reducing the accumulation of Na^+^ in maize leaves (Zhang et al., 2018; Cao et al., 2019). Generally, the inhibition of plant growth by salinity is mediated by a rapid response to changes in the external osmotic pressure, while the accumulation of Na^+^ further reduces plant growth in a slow, persistent manner (Munns and Tester, 2008). The high cellular concentration of Na^+^ subsequently induces the accumulation of reactive oxygen species (ROS), which further induces the oxidative disruption of cell membranes and damage to photosynthetic pigments (Munns, 2002; Yang and Guo, 2018).

Numerous studies have established the important roles of GB as a compatible osmolyte in plant response to abiotic stresses, including salinity, drought, and low temperature (Munns and Tester, 2008; Annunziata et al., 2019; Bai et al., 2022). Certain glycophytes, including crops of the Poaceae family, can biosynthesize and accumulate GB for adapting to salt stress (Annunziata et al., 2019). However, GB biosynthesis in plant tissues is highly energy intensive (Mäkelä et al., 1996). Therefore, the exogenous application of GB is crucial for enhancing plant tolerance to abiotic stresses, including salinity. The exogenous application of GB enhances plant stress tolerance by activating the ROS scavenging system and reducing osmotic water loss (Xing and Rajashekar, 1999; Ashraf and Foolad, 2007; Bai et al., 2022). Additionally, GB preserves the thermodynamic stability of proteins, which reverses their aggregation and helps maintain their usual functional activities. This consequently reduces the peroxidation of membrane systems and helps maintain membrane stability under saline conditions (Banu et al., 2010; Gupta and Huang, 2014). Studies conducted in the past 25 years have demonstrated that GB protects the normal structure and activity of photosynthetic oxygen center (PSII) and improves the activity of Rubisco under drought stress to alleviate the inhibition of photosynthetic capacity induced by drought stress (Papageorgiou and Murata, 1995; Huang et al., 2020). The transfer of genes responsible for GB biosynthesis from microbes and plants is another effective strategy for improving GB accumulation not only in model plants, including *Arabidopsis* sp. and tobacco, but also in other crops including rape, rice, barley, and maize (Saneoka et al., 1995; Hayashi et al., 1998; Sakamoto and Murata, 1998; Holmström et al., 2000; Huang et al., 2000; Sakamoto and Murata, 2002; Quan et al., 2004; Fujiwara et al., 2008). Genetically engineered plants carrying genes for GB biosynthesis have significant tolerance to abiotic stresses, including cold, drought, and salinity; however, the underlying reason has been mainly attributed to the function of GB as a compatible osmolyte (Chen and Murata, 2011; Annunziata et al., 2019). The additional functions of GB in regulating protein activity and metabolism are attracting increasing attention (Figueroa-Soto and Valenzuela-Soto, 2018). Recent studies have reported that GB can regulate the activity of ion channels for reducing salinity-induced cellular K^+^ efflux, enhance the activity of plasma membrane (PM) H^+^-ATPases, increase phosphate uptake, and regulate phosphate homeostasis (Wei et al., 2017; Li et al., 2019). These findings suggest that GB influences the ions transmembrane transport in plants responding to environmental stress. However, the function and mechanism of action of GB in alleviating ion toxicity, including Na^+^ homeostasis, remains to be poorly understood.

Maize is one of the leading cereal crops providing protein and calories to humans and livestock, and raw materials for industrial purposes worldwide (Nuss and Tanumihardjo, 2010). However, maize is a salinity sensitive plant, and its production is facing yield loss due to secondary salinization in intensive irrigated agricultural systems (Neves-Piestun and Bernstein, 2005; Farooq et al., 2015; Li et al., 2020). As a naturally occurring substance, GB has great potential in regulating maize salt tolerance in future. Despite the significant effects and genetic evidence of using GB for improving salt tolerance in maize, there is a scarcity of studies on the regulation of cellular Na^+^ uptake and allocation in maize, which limits the application of GB in maize plants. Therefore, this study aimed to first determine the effects of GB on the growth, photosynthesis, antioxidant capacity, and Na^+^ homeostasis in maize. Secondly, the study aimed to explore the physiological mechanism underlying the effect of GB in regulating Na^+^ homeostasis in maize cells using a hydroponic cultivation system. It is expected that the results obtained herein will improve the understanding of the regulatory mechanisms underlying GB-mediated salinity stress tolerance in maize.

## Materials and methods

### Plant cultivation and treatments

Maize inbred line ND101 (formerly known as B73-329) was used as the experimental material in this study. The selected seeds were surface sterilized with 3% H_2_O_2_ by shaking for 15 minutes, and subsequently rinsed abundantly with sterilized distilled water for five times. The seeds were then sown in quartz sand and germinated in the dark for 3 days, following which they were placed under the light with a 14 h photoperiod, a dark/light temperature cycle of 25/30°C, irradiance of 400 mmol m^−2^ s^−1^ from a high-voltage sodium lamp, and a relative humidity of 55-65%. When the tip of the second leaf grew to a length of 2 cm, the seedings were transplanted to 5 L plastic boxes filled with nutrient solution and grown under the same conditions. A total of 12 seedlings were planted in each box. As described in our previous studies, the nutrient solution comprised 0.75 mM K_2_SO_4_, 0.65 mM MgSO_4_, 2 mM Ca(NO_3_)_2_, 0.1 mM KCl, 0.25 mM KH_2_PO_4_, 0.1 mM Fe-EDTA, 1.0 μM H_3_BO_3_, 1 μM MnSO_4_, 1 μM ZnSO_4_, 0.1 μM CuSO_4_, and 0.05 μM (NH_4_)_6_MoO_24_ (Zhang et al., 2016; Zhang et al., 2020). The nutrient solution was replaced every 3 days until the plants were harvested. The following treatment groups were set up in the experiment: nutrient solution only (NS); nutrient solution + GB (GB); 125mM NaCl in nutrient solution for salt stress (SS); 125mM NaCl in nutrient solution + GB (SS + GB). For each treatment group, seven pots were arranged for replicate. Following the complete expansion of the second leaf, half of the pots were treated with nutrient solution containing 100 μM GB. After two days, 125 mM NaCl was added to half of the pots in each treatment group. The concentration and duration of treatment with GB was selected according to the results of preliminary experiments, and the concentration and duration of treatment with NaCl was based on our previous study on the same inbred line (Zhang et al., 2020). The entire treatment was continued for 13 days.

### Assay of morphological properties

In order to analyze the changes in dry matter accumulation, samples of plant shoot and root were collected at 0, 2, 4, 6, 9, and 13 days after treatment with GB (DAG). Plants were rinsed and gently washed with sterilized distilled water, following which the samples were oven-dried to a constant weight at 80°C to gain the dry weight. Seven independent plants were collected from each treatment group as biological replicates. Additionally, the height of the plants collected at 6, 9, and 13 DAG, and the length and maximum width of the third and fourth leaves were measured for calculating the leaf area at 13 DAG, as these leaves had undergone expansion during the experimental period.

### Chlorophyll fluorescence parameters assay

The photosynthetic properties and parameters of chlorophyll fluorescence were measured from the third and fourth leaves, barring the area of the veins, using MultispeQ-II (PhotosynQ, USA) with corrections by LI-6400 (LI-COR, USA) and PAM-MiniII (WALZ, German). The maximal quantum yield of photosystemII (Fv/Fm), quantum efficiency of photosystem II (ΦPSII), and non-photochemical quenching (ΦNPQ), and SPAD were simultaneously recorded using MultispeQ. The parameters were determined in triplicate from each leaf, and five independent plants were selected from each treatment group as biological replicates.

### Cell membrane stability and antioxidant activity assay

Damage of cell membrane stability was represented by leaf electrolyte leakage and the malondialdehyde (MDA) content. Leaf electrolyte leakage determined with fresh collected leaf samples by the protocols described by Verslues et al. (2006), and the final conductivity of the solution was recorded after autoclaving to represent the electrolyte content in leaves. The third and fourth leaves were collected and rapidly frozen in liquid nitrogen and stored at -80°C until determination of MDA content and antioxidases activity. The determination of MDA content was produced by the thiobarbituric acid (TBA) reaction as described by de Azevedo Neto et al. (2006). In brief, 0.5 g leaf sample was homogenized in 5 mL of 0.1% (w/v) trichloroacetic acid (TCA) solution. Then, the homogenate was centrifuged at 10000 ×g for 20 min and 1 mL of the supernatant was added to 2 mL of 0.5% (w/v) TBA in 20% TCA. The mixture was incubated in boiling water for 30 min, and the reaction stopped by ice bath. The absorbance of the supernatant was recorded at 532 nm and 600 nm after centrifuging at 10,000 ×g for5 min.

The antioxidant enzymes were extracted according to the method described by Muhammad Ali et al. (Ali et al., 2021). Briefly, the leaf samples were ground with 5 mL 50 mM phosphate buffer solution (pH 7.0), 0.2 mM EDTA, and 10 mM MgCl_2_) at 4°C. The enzyme extract was subsequently purified by centrifugation at 5000 × g for 20 min for obtaining the supernatant, which was used for the enzyme assays. The activity of superoxide dismutase (SOD) was measured using the nitroblue tetrazolium (NBT) method and the absorbance was measured at 560 nm (Dhindsa et al., 1982). The peroxidase (POD) activity was measured using guaiacol and H_2_O_2_ at a wavelength of 470 nm (Zaharieva et al., 1999), while the activity of catalase (CAT) was measured at a wavelength of 240 nm using H_2_O_2_ (de Azevedo Neto et al., 2006).

### Determination of Na^+^, K^+^ and glycine betaine content

Dry samples of root and shoot were ground into a fine powder, following which 0.2 g of the samples were extracted with 1 M HCl with shaking for 12 h at 28°C and 200 rpm. The content of Na^+^ and K^+^ in the roots and shoots were analyzed with an atomic absorption spectrophotometer (SpectAA-50/55, Varian, Australia), as previously described (Mao et al., 1985; Peng et al., 2008). The accumulation of Na^+^ and K^+^ in the samples was calculated from their concentration and biomass. Five independent plants were selected from each treatment group as biological replicates.

Determination of GB content was carried out by Reinecke salt method (Li and Zhu, 2015). 0.3 g of the powder sample of each treatment group was thoroughly incubated with distilled water to extract betaine with shaking for 24 h at 200 rpm and 28°C. After centrifuging at 5000 × g for 15 min, the supernatant was pre-cooled at 4°C for 30 min and then mixed with Reinecke salt (pH=1) to generate red precipitates for 4 h at 4°C. The precipitates were rinsed three times with 99% pre-cooled diethyl ether and then redissolved in 70% acetone solution to 10 ml. The absorbance was measured at 525 nm using spectrophotometer. A calibration curve was made using known concentration of GB following the above methods and GB content was expressed as μmol g^-1^ FW.

### Cellular Na^+^ imaging and measurement of Na^+^ content with confocal laser scanning microscopy

The cellular Na^+^ content of 9-day NaCl-treated leaves was determined using the Na^+^ indicator dye, CoroNa Green (Invitrogen, United Kingdom). Sample collection and measurement was performed as previously described by Zhang et al. (2020) with some modifications. The lower epidermis of the third leaf was peeled off to expose the mesophyll cells, which were allowed to adhere to the staining solution. After staining by incubation with 20 µM CoroNa™ Green and 0.5 M mannitol for 1 hour, 20 µM FM4-64 was added for co-staining and the cells were incubated for 1 hour. The excitation wavelength of CoroNa™ Green is 488 nm and the detection wavelength ranges between 510 and 520 nm, while the detection wavelength of FM4-64 ranges between 610 and 630 nm. The concentration of Na^+^ was determined by calculating the mean intracellular fluorescence intensity of CoroNa™ Green. We set five biological repeats for each treatment group from independent plants The mean values of the observations obtained from 10 cells in each biological repeats of each treatment group represented the cellular Na^+^ concentration.

### Protoplast isolation

Protoplasts were isolated from the leaves according to the method described by Yu et al. (2021). Briefly, the plants were grown under dark conditions at 25°C in small pots, with four plants per pot. Seeds were soaked in 100 μM GB for 12h, and GB was continually added for growth. The plants were treated with 250 mM NaCl for two days since the 9^th^ day after emergence, following which the second leaves were removed for isolating the intact protoplasts. Briefly, 50 mL of enzyme solution (0.4 M mannitol, 20 mM MES (pH=5.7), 20 mM KCl, 1.5% (w/v) Onozuka R10, and 0.4% Macerozyme R10) was kept at 55°C for 10 min. The solution was then placed at 4°C and stirred for 10 min, following which 0.5 ml of 10 mM CaCl_2_ and 0.05 g BSA were added. The second leaves were cut into 0.5-1 mm strips with a razor blade and gently immersed in the enzyme solution and incubated under vacuum (100 kPa) for 30 min in the dark. The solution was incubated in the dark for 4 h with continuous shaking for 50 rpm at 28°C. Then, 25 mL of washing solution (0.4 M mannitol and 10 mM MES (pH=5.7)) was additionally added, following which the enzyme solution containing protoplasts was filtered through a 100 μm nylon mesh. The solution was centrifuged at 100 ×g for 5 min and the supernatant was discarded to obtain the precipitated protoplasts. The pellet was resuspended in 40 ml of washing buffer and the centrifugation was repeated. These resuspended protoplasts stood on ice temporarily and would be used for NMT assay and vacuole isolation in no more than 2h.

### Non-invasive micro-test technology

The net Na^+^ fluxes from the protoplasts in the roots and leaves were measured using NMT (Younger USA, LLC, MA, USA). Prior to measuring the net Na^+^ fluxes from the roots, the seedlings were treated with 100 µM GB for two days, following treatment with 125 mM NaCl solution for an additional two days. The Na^+^ fluxes at the primary root meristem zone (approximately 700 μm from the root tip) were measured. The procedure used for measuring Na^+^ flux is described hereafter. Briefly, 250 mM NaCl backfilling solution was filled into the pre-pulled and salinized micro sensor (⌀ 4.5 ± 0.5 µm, XY- CGQ-01) to a length of 1.0 cm, following which 50 μm LIXs was filled into the tip of the micro sensor. Then, 5 mM NaCl and 0.5 mM NaCl was added for calibration, following which the roots were incubated with 1 mM NaCl for measuring Na^+^ flux over a duration of 10 minutes (Cao et al., 2020). The net Na^+^ fluxes from the protoplasts were measured using a 1.2 ± 0.5 µm micro sensor. Following regulation, the protoplasts were incubated in 1 mM NaCl and 500 mM mannitol, and the Na^+^ flux was recorded for 10 minutes (Zhang et al., 2011). The osmotic pressure of the protoplasts was maintained using 500 mM mannitol. The protoplasts were also pretreated with 500 µM sodium orthovanadate, a specific PM H^+^-ATPase inhibitor, and with 1μM Amiloride, a specific PM Na+/H+ antiporter inhibitor, for 30 min prior to flux measurement. The data were measured and calculated using imFluxes 1.0 (Xuyue technology Ltd., China) according to the tutorials provided by Xuyue.

### Measurement of activity of vacuolar (V)-Na^+^/H^+^ antiporter

The activities of Na^+^/H^+^ antiporters were determined in this study, as described hereafter. Briefly, intact vacuoles were isolated from plant leaves according to the plant vacuoles isolation protocol (Robert et al., 2007), and 50 μg of vacuolar membrane protein was used for determining the activity of the Na^+^/H^+^ antiporters. Na^+^/H^+^ exchange activity was calculated based on Na^+^-induced dissipation (Qiu et al., 2002). The fluorescence due to quinacrine was measured using a spectrofluorometer (Hitachi F-7500 imager, HITACHI, Japan).

### RNA extraction and real-time quantitative polymerase chain reaction

The total RNA of the third and fourth leaves was extracted using an EASY spin rapid plant RNA extraction kit (Aidlab, China). Reverse transcription (RT) was then performed using an M5 Sprint qPCR RT kit with genomic DNA remover, following which real-time qPCR was performed using TB Green^®^ Premix Ex TaqTM II (Takara, China) on a 7500 fast real-time PCR system (Applied Biosystems, Foster City, CA, United States). The *ZmUBC* ubiquitin ligase gene (ubiquitin C) of maize was selected as the internal control, and relative gene expression was calculated using the 2^-△△CT^ method. Three independent plants were selected from each treatment group as biological replicates. The primers used for real-time qPCR amplification are enlisted in **Supplementary Table S1** (Qin et al., 2019; Cao et al., 2020; Zhang et al., 2020; Zhou et al., 2022).

### Statistical analyses

Statistical analyses were conducted with SAS software (V8, SAS Institute Inc., Cary, NC, United States). Fisher’s protected least significant difference (LSD) test was used to perform multiple comparisons with corrections. P-values < 0.05 were considered to be significant.

## Results

### GB promoted growth in maize under NS and SS conditions

**Figure 1:**
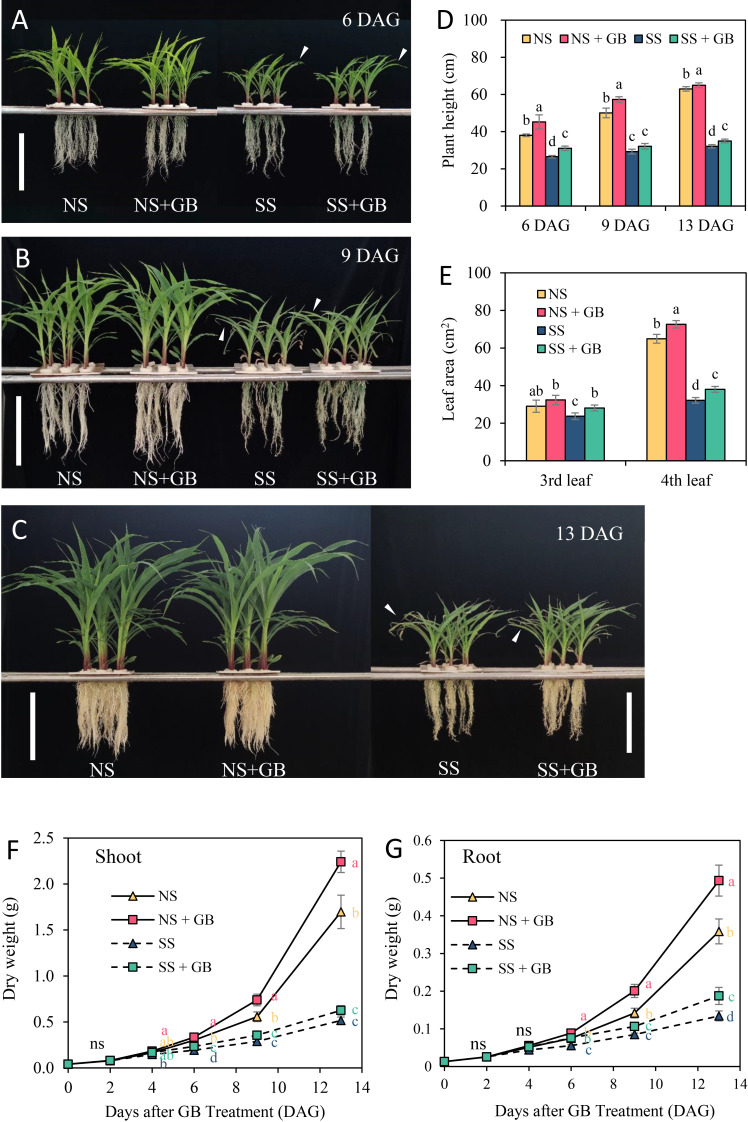
Effects of GB on the growth of plants under non-stressed (NS) and salinity-stressed (SS) conditions. Phenotypes of plants treated with 0 and 100 µM GB under NS and SS conditions at **(A)** 6 days, **(B)** 9 days and **(C)** 13 days after GB treatment. White vertical bar = 20 cm. **(D)** Heights of plants treated with 0 and 100 µM GB under NS and SS conditions. **(E)** Area of third and fourth leaves at 13 days of treatment. Dry weight of **(F)** shoots and **(G)** roots for each of the treatment groups. The values indicate the mean, and the vertical error bars indicate the standard deviation (n = 7). The different letters in each panel represent significant differences on the same day, as determined with Fisher’s protected LSD test at P < 0.05. ns, no significance.

GB promoted the growth of plant shoots and roots under both NS and SS conditions, and mitigated salinity-induced leaf senescence (**Figure 1**). A concentration of 100 μM GB was found to be optimal for increasing the dry weight of maize under NS conditions; however, an increase in the concentration of GB to 1 mM had obvious negative effects on the plants (**Supplementary Figure S1**). GB treatment with optimal concentration significantly increased the endogenous GB content in maize leaf by 19.3% to 42.9% after GB treatment (DAG) under NS, and 125 mM NaCl treatment increased GB content in maize plant at 6 DAG, that is 4 days after treatment with NaCl (**Supplementary Figure S2**). Compared with non-saline conditions, salt significantly inhibited plant growth. However, treatment with GB increased plant height by 8.9%-17.0%, and the leaf area of the third and fourth leaves increased by 18.5% and 18.1%, respectively, in SS plants when harvested at 13 days after GB treatment (DAG), that is, 11 days after treatment with NaCl (**Figures 1D, E**). There was a significant difference between treatments with respect to the biomass accumulated in shoots at 4 DAG and in roots at 6 DAG. Finally, treatment with GB increased the accumulation of biomass in shoots by 13.6% and 23.3% under NS and SS respectively, and in roots by 18.1% and 39.5%, respectively (**Figures 1F, G**). These results suggested that GB effectively improved plant growth under NS and SS conditions; however, the effects of GB on improving plant growth were more pronounced under SS conditions than under NS conditions.

### GB preserved photosynthetic activity, improved the antioxidant capacity and maintained membrane stability under salt stress

**Figure 2:**
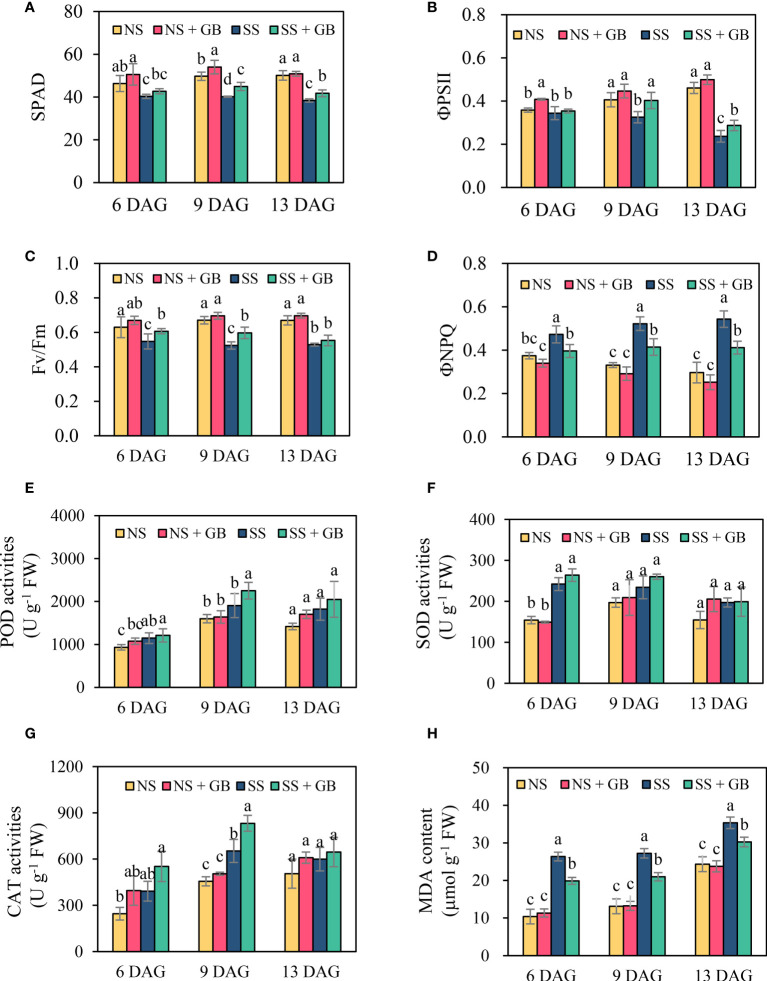
Effects of GB on the maize leaf photosynthesis capacities, antioxidases activity and cell membrane stability under non-stressed (NS) and salinity-stressed (SS) conditions. **(A)** SPAD, **(B)** quantum efficiency of photosystem II (ΦPSII), **(C)** maximal quantum yield of photosystem II (Fv/Fm), **(D)** non-photochemical quenching (ΦNPQ), **(E)** peroxidase (POD) activity, **(F)** superoxide dismutase (SOD) activity, **(G)** catalase (CAT) activities, and **(H)** malondialdehyde (MDA) content. The values indicate the mean, while the vertical error bars indicate the standard deviation (n = 5). The different letters in each panel represent significant differences determined with Fisher’s protected LSD test at P < 0.05.

As expected, treatment with GB improved the photosynthetic capacity of leaves under both NS and SS conditions (**Figures 2A–D**, and **Supplementary Figure S3**). Under normal conditions, exogenous GB increased the SPAD value, ΦPSII, and Fv/Fm, and reduced the ΦNPQ; however, the differences between GB-treated and untreated groups were not significant. Salt stress obviously induced leaf senescence and damaged the photosynthetic structures. The application of GB under SS conditions increased the SPAD value of the third leaf by 4.2%, 11.8%, and 3.6% at 6, 9, and 13 DAG, respectively. Similar results were obtained for ΦPSII and Fv/Fm. GB increased ΦPSII by 18.4%, 23.9%, and 36.9% in the third leaf at 6, 9, and 13 DAG, respectively, and by 26.1% and 45.7% in the fourth leaf at 9 and 13 DAG, respectively. Additionally, ΦNPQ was significantly reduced by 18.8%, 20.6%, and 18.7% in the third leaf at 6, 9, 13 DAG, respectively, and by 19.4% and 30.5% in the fourth leaf at 9 and 13 DAG, respectively.

Generally, salt stress and treatment with GB increased the activity of antioxidant enzymes, POD, SOD, and CAT. The activity of the antioxidant enzymes exhibited distinct tissue-specific and time-specific patterns (**Figures 2E–G**, and **Supplementary Figure S4**). The POD activity in the roots was approximately 20-fold that in the shoots. There was no significant difference in the POD activity of GB-treated and control groups under NS, while treatment with GB significantly increased the POD activity under SS conditions. The activity of SOD was similar in the shoots and roots, and significant differences were observed between the GB-treated and control groups at 6 DAG under NS conditions. Treatment with GB significantly increased the CAT activity in shoots and the difference increased with the duration of salt stress.

Cell malondialdehyde (MDA) content indicated the damage of stress on membrane stability. **Figure 2H** depicted leaf MDA accumulation in response to salt and GB treatment. With the increase of growth time, the third leaf MDA content increased. Salt stress significantly stimulated MDA production in leaf cells. Treatment with GB remarkably alleviated MDA accumulation by 14.3%-25.6% than the control under SS condition, while the difference between control and GB was not significant under NS condition. Consistent with results of cell MDA accumulation, GB treatment reduced leaf cell electrolytes leakage under SS condition (**Supplementary Figure S5**).

### Exogenous GB decreased Na^+^ accumulation and Na^+^/K^+^ ratio

**Figure 3:**
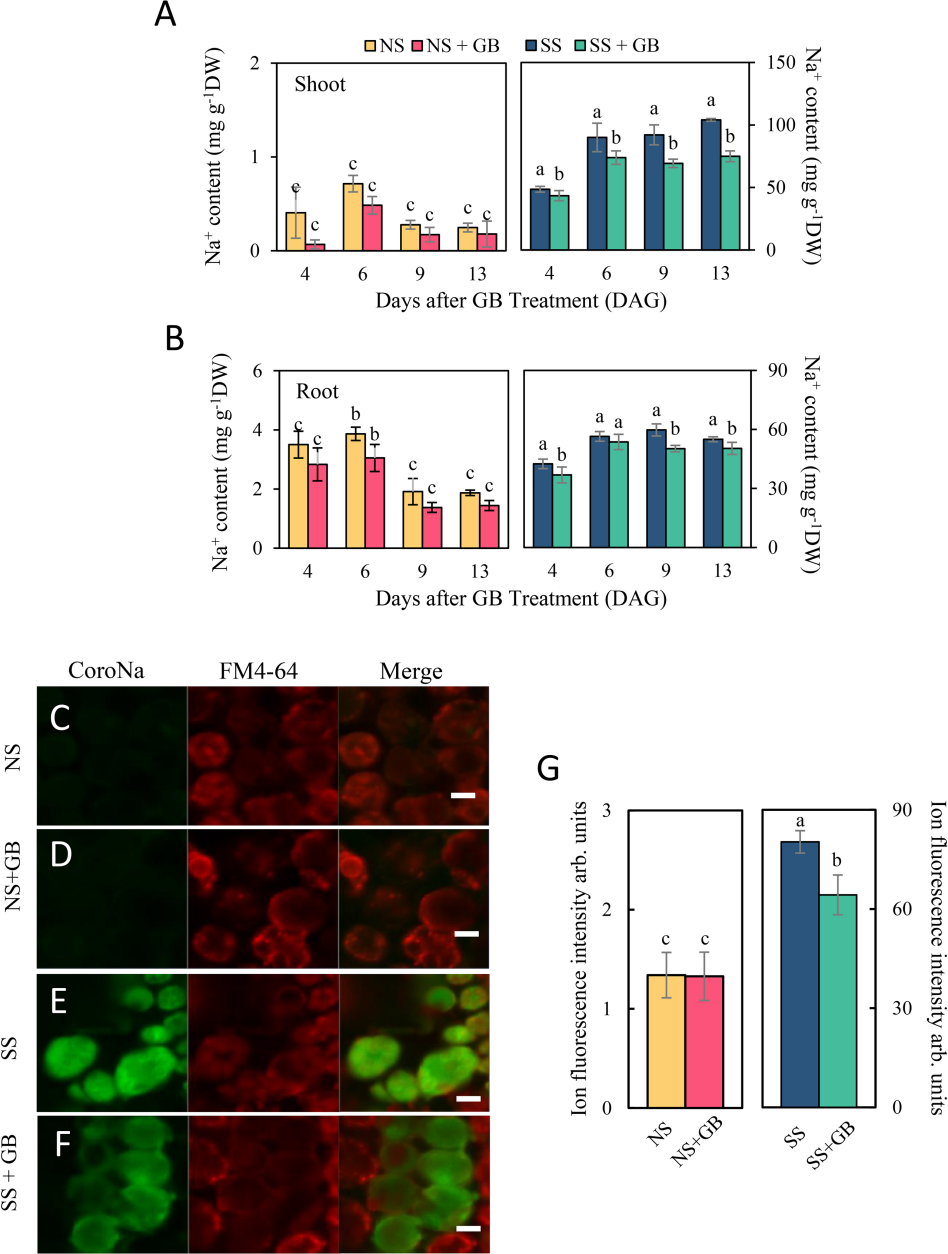
Effects of GB on maize plant and cellular Na^+^ accumulation under non-stressed (NS) and salinity-stressed (SS) conditions. **(A)** Concentration of Na^+^ in the shoots and **(B)** roots. The values indicate the mean, while the vertical error bars indicate the standard deviation (n = 5). The different letters in each panel represent significant differences determined with Fisher’s protected LSD test at P < 0.05. **(C-F)** depicted effects of GB on the fluorescence intensity of cellular Na^+^ after 3 days of treatment with NaCl. Na^+^ was stained using CoroNa™ Green, while the cell membrane was stained with FM4-64. **(C)** control under NS condition, **(D)** GB-treated group under NS condition, **(E)** control under SS condition, and **(F)** GB-treated group under SS condition. White horizontal bar = 10 μm. **(G)** Statistic of fluorescence intensity of cellular Na^+^. The values indicate the mean, while the vertical error bars indicate the standard deviation (n = 50). The different letters on the bar represent significant differences determined with Fisher’s protected LSD test at P < 0.05.

As depicted in **Figure 3** and **Supplementary Figure S6**, treatment with NaCl significantly increased the concentration of Na^+^ and decreased the concentration of K^+^ in plants. Following the application of GB, the concentration of Na^+^ in the roots and shoots decreased by 5.0%-23.9% and 10.6%-24.7%, respectively. However, there was no difference in the K^+^ concentration of the roots and shoots in response to treatment with 125 mM NaCl, with or without GB. Even under NS conditions, exogenous GB had obvious effects on decreasing the Na^+^ concentration in both shoots and roots by reducing the Na^+^ content. Additionally, the Na^+^/K^+^ ratio in the roots and shoots decreased by 12.7%-28.3% and 9.6%-32.5%, respectively, following the application of GB, which was primarily attributed to the reduction in Na^+^ content. These observations corroborated with the fluorescence signals from the mesophyll cells of the third leaf. Salt stress significantly enhanced the green fluorescent signal due to Na^+^ in mesophyll cells, while the fluorescence signal was reduced following the application of GB (**Figures 3C-G**).

### Exogenous GB enhanced Na^+^ efflux through PM H^+^-ATPases

**Figure 4:**
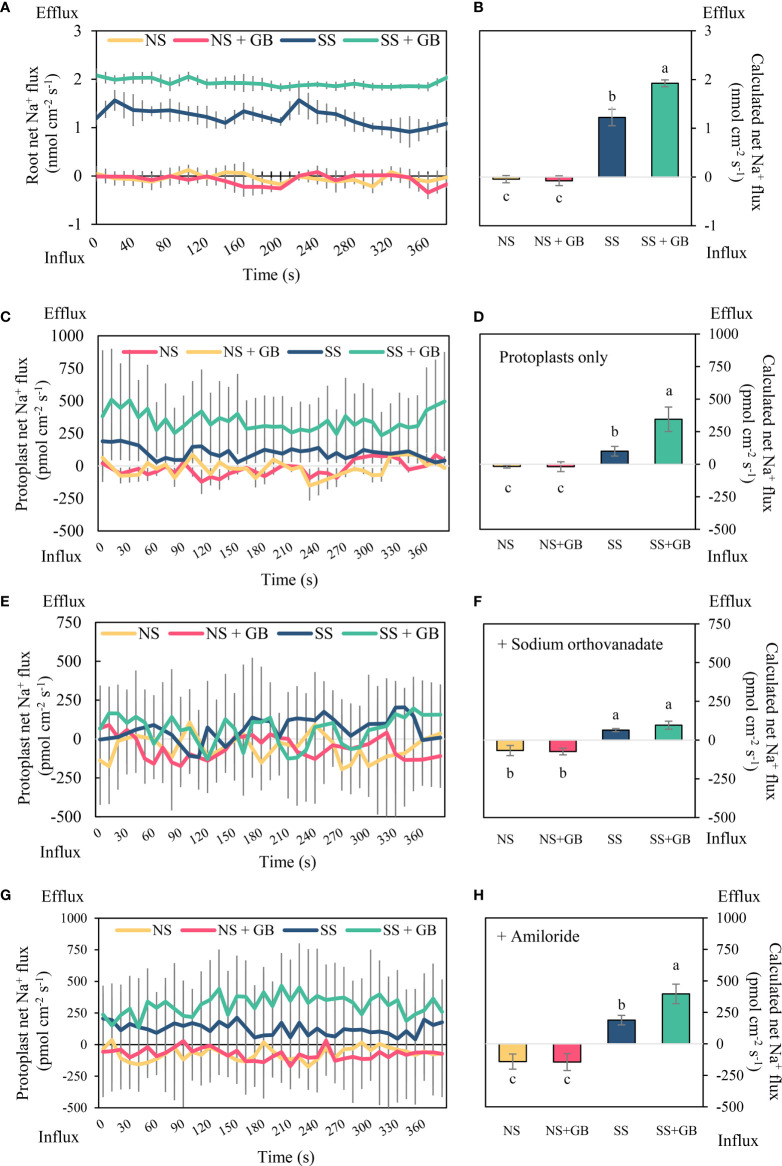
Effects of GB on Na^+^ flux in roots and protoplasts with membrane ion transporter inhibitors under non-stressed (NS) and salinity-stressed (SS) conditions. **(A)** Real-time net flux of Na^+^ in roots and **(B)** corresponding calculated mean Na^+^ flux rates during measurement period. **(C)** Real-time net Na^+^ flux in leaf protoplasts and **(D)** corresponding calculated mean Na^+^ flux rates during measurement period. **(E)** Real-time net Na^+^ flux in leaf protoplasts with sodium orthovanadate treatment, and **(F)** corresponding calculated mean Na^+^ flux rates during measurement period. **(G)** Real-time net Na^+^ flux in leaf protoplasts with amiloride treatment, and **(H)** corresponding calculated mean Na^+^ flux rates during measurement period. The values indicate the mean, while the vertical error bars indicate the standard deviation (n = 5). The different letters in each panel represent significant differences determined with Fisher’s protected LSD test at P < 0.05.

The Na^+^ fluxes in the roots and leaves protoplasts were determined using NMT. Treatment with GB significantly improved Na^+^ flux properties in the roots to 1900 pmol cm^−2^ s^−1^ compared to that of the control following treatment with 125 mM NaCl for two days (**Figures 4A, B**). Similar to the results observed in the roots, the leaf protoplasts exhibited an outward rectification with a mean value of 100 pmol cm^−2^ s^−1^ under SS conditions, while protoplasts treated with exogenous GB exhibited enhanced Na^+^ efflux with a value of 350 pmol cm^−2^ s^−1^. Additionally, there were no significant differences in Na^+^ fluxes between GB-treated and NS groups (**Figures 4C, D**). The Na^+^ efflux of GB-treated protoplasts was immensely reduced by 72% following the addition of 0.5 mM sodium orthovanadate, a specific inhibitor of PM H^+^-ATPases, compared with that of the control group under salt stress (**Figures 4E, F**). While pretreating with amiloride, the specific inhibitor of SOS1, significantly reduced the Na^+^ efflux under SS than that without any inhibitor treatment, but amiloride treatment could not mitigate the significant increasing trend induced by GB treatment (**Figures 4G, H**). These results indicated that exogenous GB enhanced Na^+^ efflux primarily through PM H^+^-ATPases.

### Effects of GB on the activities of vacuolar H^+^-ATPases and NHX

**Figure 6:**
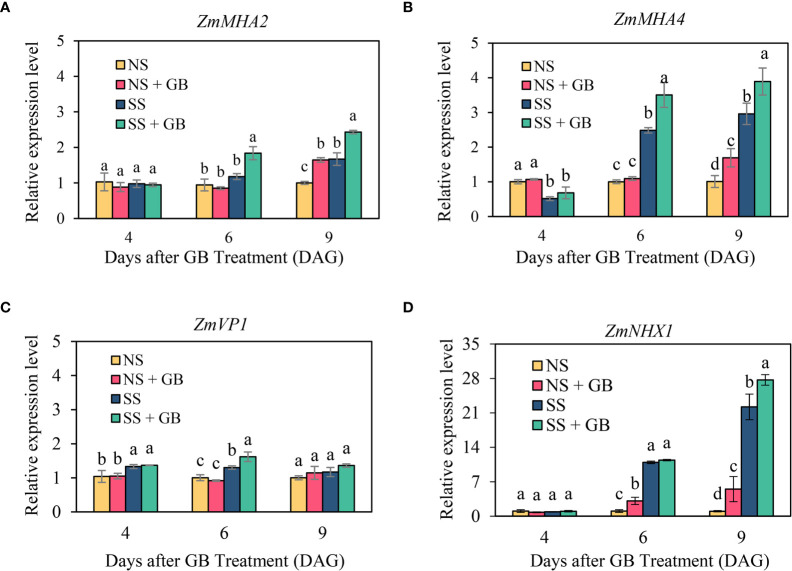
Effects of GB on the expression of **(A)** ZmMHA2, **(B)** ZmMHA4, **(C)** ZmVP1, and **(D)** ZmNHX1 in the leaves of maize plants under non-stressed (NS) and salinity-stressed (SS) conditions. The values indicate the mean, while the vertical error bars indicate the standard deviation (n = 3). The different letters in each panel represent significant differences determined with Fisher’s protected LSD test at P < 0.05

As vacuolar (V type) H^+^-ATPases and NHX play important roles in plant salt response, we investigated the activity of the vacuolar Na^+^/H^+^ antiporters of plants treated with 100 µM GB for eleven days and 250 mM NaCl for two days (**Figure 6**). The results indicated that salt stress increased the activity of maize vacuolar NHX and V type H^+^-ATPase by 29.1% and 15.8% respectively. The plants that had been treated with GB exhibited markedly increased V-type Na^+^/H^+^ antiporter activity under SS conditions by 87%, while there was no obvious effect on the activity of V type H^+^-ATPases.

### Exogenous GB modulated the expression of ion transporter related genes

**Figure 5:**
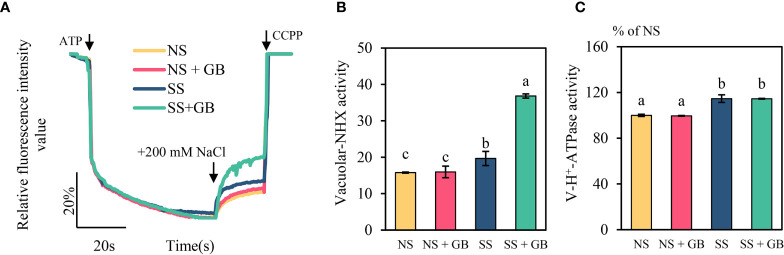
Effects of GB on the activity of vacuolar H^+^-ATPase and NHX. **(A)** Activity of vacuolar Na^+^/H^+^ antiporters, **(B)** mean rate of Na^+^/H^+^ antiporter activity over the duration of measurement, and **(C)** H^+^-ATPase activity. The values indicate the mean, while the vertical error bars indicate the standard deviation (n = 5). The different letters in each panel indicate significant differences determined with Fisher’s protected LSD test at P < 0.05.

As depicted in **Figure 5**, salt stress and exogenous GB significantly affected the transcription levels of the PM H+-ATPase genes, V type H+-ATPase and vacuolar Na^+^/H^+^ exchanger gene, in maize leaves, although salt induced upregulation of gene expression was not significant on the 2^nd^ day after salt treatment, i.e., 4 DAG. Salt stress significantly upregulated the transcription of the reported PM H^+^-ATPase gene, *ZmMHA4* and *ZmMHA2* after 6 DAG; but the transcriptional upregulation of *ZmMHA2* was lower than that of *ZmMHA4*. Under NS, GB significantly improved the expression of ZmMHA2 and ZmMHA4 on the 9 DAG only, while GB treatment significantly increased the expression of *ZmMHA4* by 44% and 32% under SS on the 6 DAG and 9 DAG respectively. Consistent with the results of vacuolar H^+^-ATPase and NHX activity, GB did not significantly alter the transcription level of the V type H^+^-ATPase gene, *ZmVP1*. However, the transcription level of the Na^+^/H^+^ exchanger gene, *ZmNHX1*, was significantly upregulated by salt treatment on the 6 DAG, i.e. 4 days after salt treatment in the leaves. GB treatment significantly increased the expression of ZmNHX1 under NS on the 6 DAG and 9 DAG, while the difference between GB and non-GB treated plants was only significant on the 9 DAG under SS.

## Discussion

The exogenous application of GB and genetic modifications for enhancing endogenous GB biosynthesis are effective measures for overcoming various environmental stresses in plants. The primarily mechanisms underlying the effect of GB in mitigating plant stress include osmotic adjustment, alleviation of photosynthesis and respiration, gene regulation, and cellular/subcellular protection (Huang et al., 2000; Apse and Blumwald, 2002; Ashraf and Foolad, 2007; Munns and Tester, 2008; Chen and Murata, 2011; Wei et al., 2017; Li et al., 2019; Ayub et al., 2022). Previous studies have reported that the function of GB in improving the salt tolerance of plants is primarily attributed to osmotic adjustment and stabilization of the antioxidant system in many plants (Saneoka et al., 1995; Munns and Tester, 2008; Farooq et al., 2015; Figueroa-Soto and Valenzuela-Soto, 2018; Annunziata et al., 2019). The results of this study similarly demonstrated that GB aids in the maintenance of photosynthesis and antioxidant systems in maize under conditions of salinity (**Figure 2** and **Supplementary Figures S3**, **S4**). Moreover, this study extended the knowledge of GB playing roles in regulating cell Na^+^ homeostasis.

### GB maintained leaf photosynthetic capacity and reduced ROS damage in response to salinity stress

GB significantly alleviated the senescence of expanded leaves (**Figure 1**), which was consistent with the SPAD value and fluorescence properties of chlorophyll following treatment with GB. Previous studies on the GB biosynthesis gene modified rice and tobacco reported similar results that enhancing GB biosynthesis improve photosynthesis by altering the parameters of photochemical quenching, including Fv/Fm, ΦPSII, and qP, except for the net photosynthesis rate (Sakamoto and Murata, 1998; Yang and Lu, 2005). The ΦNPQ provides a measure of non-photochemical quenching, which is closely associated with the onset of the harmless dissipation of excess energy in the pigment bed of light-harvesting complexes in the form of heat (Baker, 2008; Li et al., 2020). In this study, we observed that the ΦNPQ was more sensitive to salt stress than other parameters of chlorophyll fluorescence, and GB had significant effects on reducing the ΦNPQ under conditions of salinity. Similar results were reported by studies on improving the salt tolerance of maize using bio-stimulants (Li et al., 2020).

Due to the salt stress was kept for more than weeks, the inhibition of photosynthetic capacity by salt stress could be mainly attributed to the cellular accumulation of Na^+^, which triggered ROS-mediated damages to the membrane structure. The positive effects of GB on ROS scavenging in both GB biosynthesis gene modified and exogenous GB-treated plants are well documented (Saneoka et al., 1995; Munns and Tester, 2008; Farooq et al., 2015; Figueroa-Soto and Valenzuela-Soto, 2018; Annunziata et al., 2019), which are consistent with the results obtained in this study (**Figures 2E-G** and **Supplementary Figure S4**, **S5**). Besides, enhancing GB biosynthesis could not only maintained the activity of antioxidases, but numerous studies have also demonstrated that GB could stabilize protein structure, including the enzymes associated with sugar and amino acid metabolism in transgenic maize than in the control plants under environmental stress, like drought and low temperature (Sakamoto and Murata, 2002; Quan et al., 2004). Meanwhile, GB could maintain the membrane stability and reduce the oxidant damage by binding to the phospholipid molecules (Figueroa-Soto and Valenzuela-Soto, 2018; Annunziata et al., 2019). Similar results, that GB alleviated salt induced MDA accumulation and electrolytes leakage, were obtained in this study. (**Figure 2H** and **Supplementary Figure S5**). As summarized in **Figure 6**, previous study and this study have well reported the effects of GB on reducing osmotic stress and oxidant damage. The function of GB in regulating Na^+^ homeostasis for mitigating ion toxicity under salt stress has been poorly investigated.

### GB Reduced Na^+^ accumulation to improve maize salt tolerance

The maintenance of cellular ion homeostasis, especially a suitable Na^+^/K^+^ ratio, is an important adaptive trait for plants growing in a saline environment and is studied by fluorescence staining. It is well accepted that plants always increase K^+^ accumulation to alleviate ion toxicity induced by Na^+^ over uptake under salt stress (Yang and Guo, 2018). A recent study suggested that exogenous GB increases the salt tolerance of common bean (*Phaseolus vulgaris* L.) by limiting Na^+^ uptake or increasing the accumulation of K^+^ (Sofy et al., 2020). Previous studies reported similar results in many other plants (de la Torre-Gonzalez et al., 2018; Wei et al., 2017). However, there was few information about how GB regulated maize K^+^ and Na^+^ uptake. Our results suggested similar results that NaCl treatment decreased K^+^ content in maize plant. GB significantly reduced the Na^+^/K^+^ ratio in both roots and shoots, which was primarily attributed to the reduction in Na^+^ concentration in the roots and shoots of maize, while the concentration of K^+^ was not significantly increased in maize plants (**Figure 2** and **Supplementary Figure S5**). In a study on effects of silicon and selenium on enhancing salt tolerance of maize plants (Xu et al., 2021), it was reported that Si and Se significantly reduced Na^+^ uptake but had no significant effects on K+ accumulation, which were like our results. These indicated that there would be a complicated mechanism in plants to regulate Na^+^ and K^+^ balance under various environments, which need much further studies.

### GB reduced Na^+^ uptake mainly by regulating PM H^+^-ATPases activity

Based on the results of NMT and fluorescence assay (**Figures 3**, **4A**), we further investigated the mechanism of GB regulating cell Na^+^ homeostasis. The mechanisms for reducing cytoplasmic Na^+^ include the restriction of Na^+^ uptake, increased cellular Na^+^ efflux, and vacuolar sequestration of Na^+^ (Wu, 2018; Yang and Guo, 2018). PM H^+^-ATPases play master roles in plant growth, development, and salinity stress responses (Li et al., 2022). SOS1-mediated Na^+^ efflux requires the generation of a proton gradient by PM H^+^-ATPases in response to salt stress (Qiu et al., 2002; Yang et al., 2019). An increase in the activity of PM H^+^-ATPases increases salinity tolerance in plants (Shi et al., 2000; Yang and Guo, 2018). In this study, GB enhanced the efflux of Na^+^ from the leaf protoplasts of maize following salinity treatment by approximately 3-fold, and treatment with sodium orthovanadate, a PM H^+^-ATPase inhibitor, significantly mitigated the positive effects of GB on cellular Na^+^ efflux, while amiloride could not restrain the enhancing effects of GB on cellular Na^+^ efflux under SS (**Figures 4C–H**). This indicated that GB increased the efflux of Na^+^ from maize cells, which could be primarily attributed to the upregulated activity of PM H^+^-ATPases under saline conditions. Recent studies have suggested that GB regulates the activity of PM H^+^-ATPases and reduces heavy metal toxicity and phosphate deficiency (Li et al., 2019; Li et al., 2021). However, the increasing effects of GB on PM H^+^-ATPase activity was not significantly under NS condition, which indicated that there may be a co-effector working with GB to play this role in regulating Na^+^ efflux under SS.

**Figure 7:**
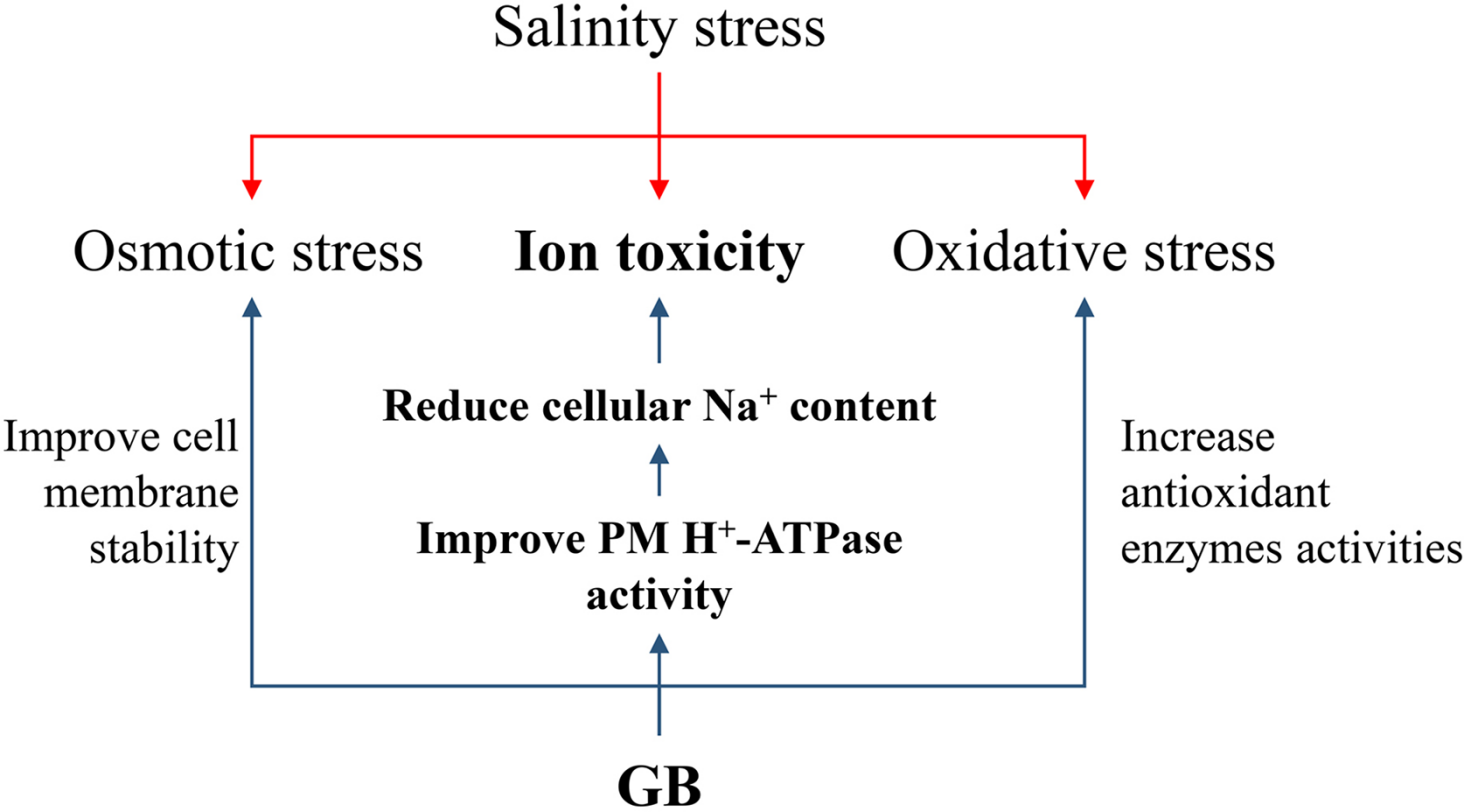
Summary of the roles of GB in regulating to improve maize salt stress tolerance. The red arrows indicated the main three categories of damages induced by salinity. The blue arrows indicated the process of GB improving maize salinity stress tolerance. The bolded letters indicated the main results that GB reduced maize cellular Na+ content by improving PM H+-ATPase activities to alleviate ion toxicity induced by salinity.

Increasing in the expression of genes encoding PM H^+^-ATPases are involved in the salt tolerance of maize (Cao et al., 2020). As expected, GB upregulated the expression of *MHA2* and *MHA4* under conditions of salinity in this study (**Figure 6**), and similar results have been reported by previous studies on tomato (Wei et al., 2017). Recent studies have demonstrated that GB regulates the expression of target proteins and modulates their role in plant metabolic processes rather than by only acting as an osmolyte (Figueroa-Soto and Valenzuela-Soto, 2018). Additionally, endogenous small molecules of plants, including γ-aminobutyric acid and free unsaturated fatty acids such as oleic acid, linoleic acid, and linolenic acid, can activate PM H^+^-ATPases by directly binding to the C-terminus of PM H^+^-ATPases (Han et al., 2017). However, the molecular mechanisms underlying the regulatory effect of GB on H^+^-ATPases and sodium transporters in the PM require further investigation. Besides, analyses of the processes of vacuolar Na^+^ sequestration revealed that GB had significant effects on the activity and gene expression of vacuolar NHX under conditions of salinity (**Figures 6**, **7**). However, the activity of V-type H^+^-ATPases was not significantly regulated by GB (**Figure 7**).

These results indicated the complexity and diversity of the regulatory mechanism of GB on membrane ion transportation in plant cells at both gene expression and protein activity level. This study provides some insights into the regulatory activity of GB on cellular Na^+^ homeostasis under salt stress, and further studies are necessary for investigating the molecular mechanism underlying the interactions between GB and target protein.

## Conclusion

The application of GB at the optimal concentration of 100 μM significantly improved photosynthesis and antioxidant activity in maize for maintaining plant growth under conditions of salinity. Compared with the control group, GB decreased the accumulation of Na^+^ in shoots and roots, which was primarily attributed to the reduced rate of Na^+^ uptake in roots and higher efflux of Na^+^ from the cells of leaves. GB significantly increased the gene expression of genes encoding PM H^+^-ATPases, PM Na^+^ transporters, and vacuolar NHX in the leaves of maize, and increased the enzymatic activity of PM H^+^-ATPases for improving cellular Na^+^ efflux. Therefore, apart from its role as an osmolyte and antioxidants activator, GB improved the salt tolerance of maize by increasing the gene expression and activity of PM H^+^-ATPases, which further alleviated Na^+^ toxicity.

## Data availability statement

The original contributions presented in the study are included in the article/**Supplementary Material**. Further inquiries can be directed to the corresponding author.

## Author contributions

MZhu, QL, YZ, and MZha designed the experiments. MZhu, QL, and YZ performed the experiments, analyzed the data, constructed the plots, and prepared the manuscript. All authors contributed to the article and approved the submitted version.

## Funding

This work was supported by the National Natural Science Foundation of China (grant numbers: 32001458 and 31930079).

## Conflict of interest

The authors declare that the research was conducted in the absence of any commercial or financial relationships that could be construed as a potential conflict of interest.

## Publisher’s note

All claims expressed in this article are solely those of the authors and do not necessarily represent those of their affiliated organizations, or those of the publisher, the editors and the reviewers. Any product that may be evaluated in this article, or claim that may be made by its manufacturer, is not guaranteed or endorsed by the publisher.
