# Supplementary material for: Glycine betaine increases salt tolerance in maize (Zea mays L.) by regulating Na+ homeostasis
Source: Front Plant Sci. 2022 Sep 30;13:978304. doi: 10.3389/fpls.2022.978304 (PMC9562920; doi:10.3389/fpls.2022.978304)

Supplementary Material

1 Supplementary Table S1: Primers used in this study.

**RT-qPCR Primers**			
Gene Name	Gene ID	Forward primer sequence (5' to 3')	Reverse primer sequence (5' to 3')
ZmUBC	Zm00001d015327	CTGGTGCCCTCTCTCCATATGG	CAACACTGACACGACTCATGACA
ZmVP1	Zm00001d015569	CTGGGACAACGCCAAGAAGT	AGCACATGCCATAGATGCTG
ZmMHA2	Zm00001d002006	TACTGAGCCAGGTCTTAGCG	CCAGATCAACGCAATGAGCA
ZmMHA4	Zm00001d026490	TCCTCAAGTTCCTCGGGTTC	GGTGGAGTTGATGACCAGGA
ZmNHX1	Zm00001d048732	GTTAACGAGTCCATCACCGC	CCGGCATTGAAGATGATGGG

2 Figure S1 The phenotype on maize under different GB concentration

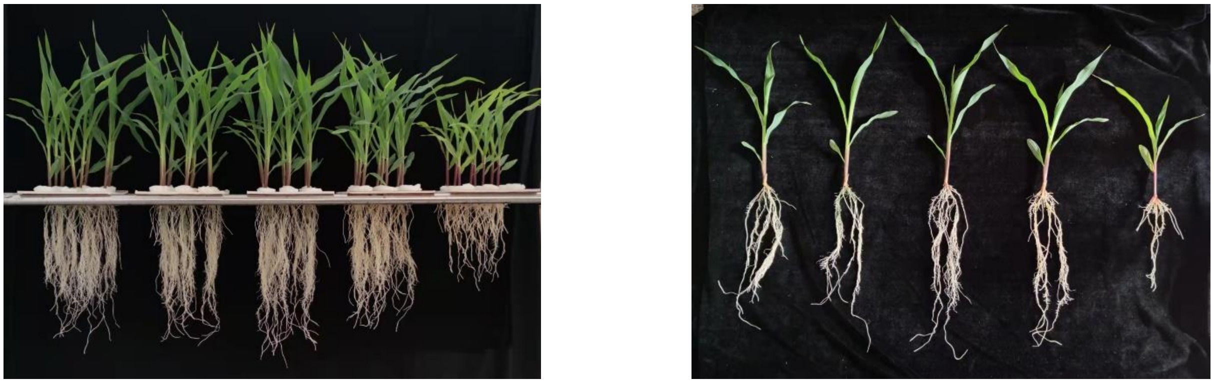

CK 10μM 100μM 1mM 10mM GB

CK 10μM 100μM 1mM 10mM GB

Figure S2 Effects of GB on the endogenous betaine content in maize leaves under non-stressed (NS) and salinity-stressed (SS) conditions. The values indicate the mean, while the vertical error bars indicate the standard deviation (n = 5). The different letters in each panel represent significant differences determined with Fisher's protected LSD test at $P < 0.05$.

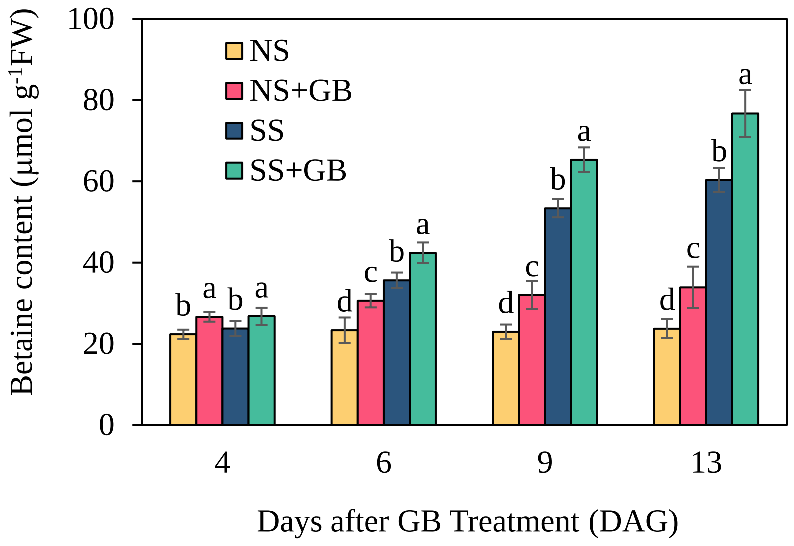

4 **Figure S3 Effects of GB on the photosynthesis capacities and parameters of the fourth leaf chlorophyll fluorescence in maize.** Values of (A) SPAD, (B) quantum efficiency of photosystem II (ΦPSII), (C) maximal quantum yield of photosystemII (Fv/Fm), and (D) non-photochemical quenching (ΦNPQ) of maize leaves under non-stressed (NS) and salinity-stressed (SS) conditions. The values indicate the mean, while the vertical error bars indicate the standard deviation (n = 5). The different letters in each panel represent significant differences determined with Fisher's protected LSD test at $P < 0.05$.

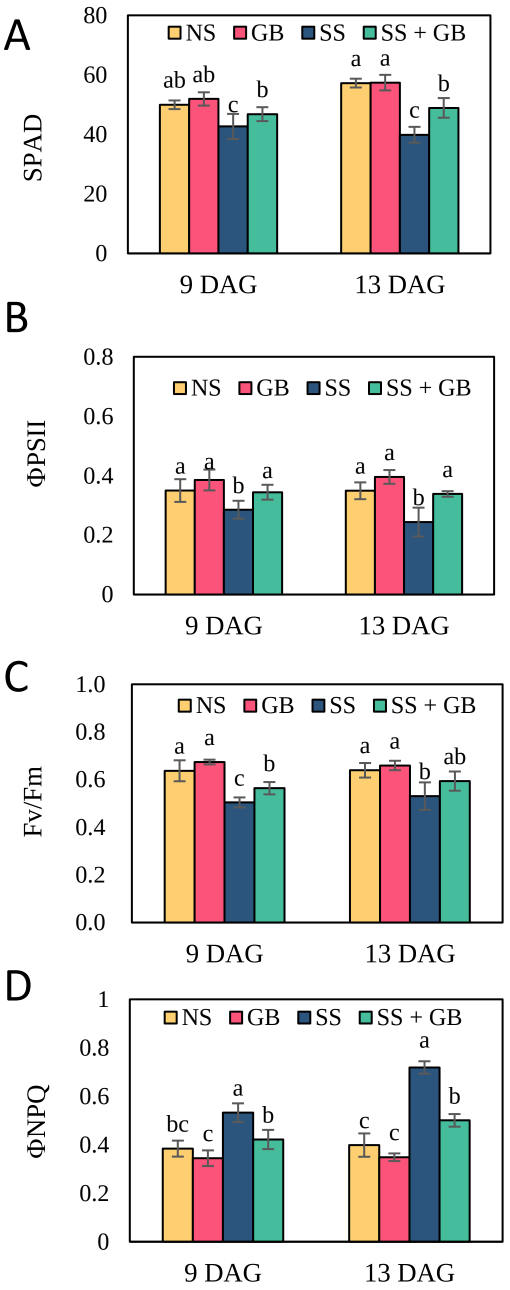

Figure S4 Effects of GB on the activities of (A) POD, (B) SOD, and (C) CAT in maize root under non-stressed (NS) and salinity-stressed (SS) conditions. The values indicate the mean, while the vertical error bars indicate the standard deviation (n = 3). The different letters in each panel represent significant differences determined with Fisher's protected LSD test at P < 0.05.

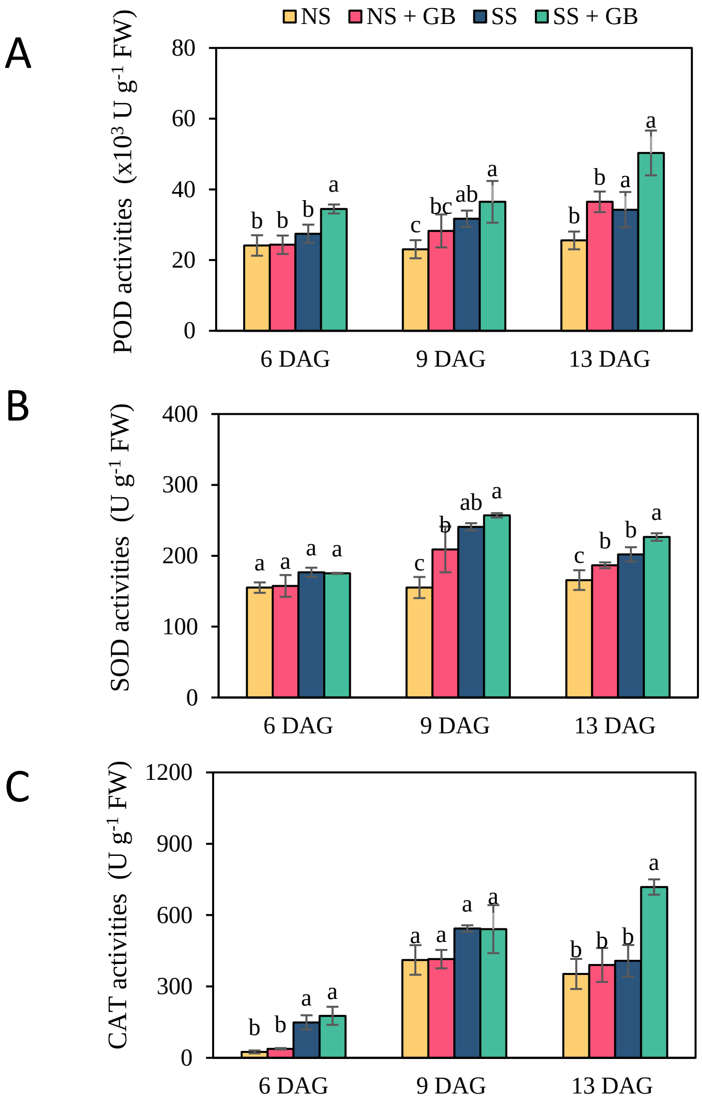

6 **Figure S5 Effects of GB on electrolytes leakage in maize leaf under non-stressed (NS) and salinity-stressed (SS) conditions.** The values indicate the mean, while the vertical error bars indicate the standard deviation (n = 3). The different letters in each panel represent significant differences determined with Fisher's protected LSD test at P < 0.05.

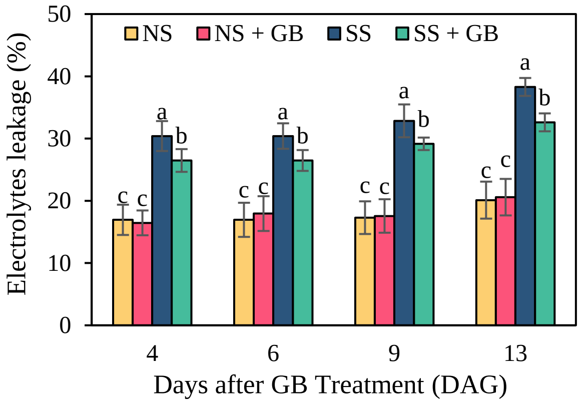

Figure S6 Concentration of (A) K^+, (B) Na^+/K^+ ratio in shoots and concentration of (C) K^+, and D) Na^+/K^+ ratio in the roots of GB-treated and control plants under non-stressed (NS) and salinity-stressed (SS) conditions. The values indicate the mean, while the vertical error bars indicate the standard deviation (n = 5). The different letters in each panel represent significant differences determined with Fisher's protected LSD test at $P < 0.05$.

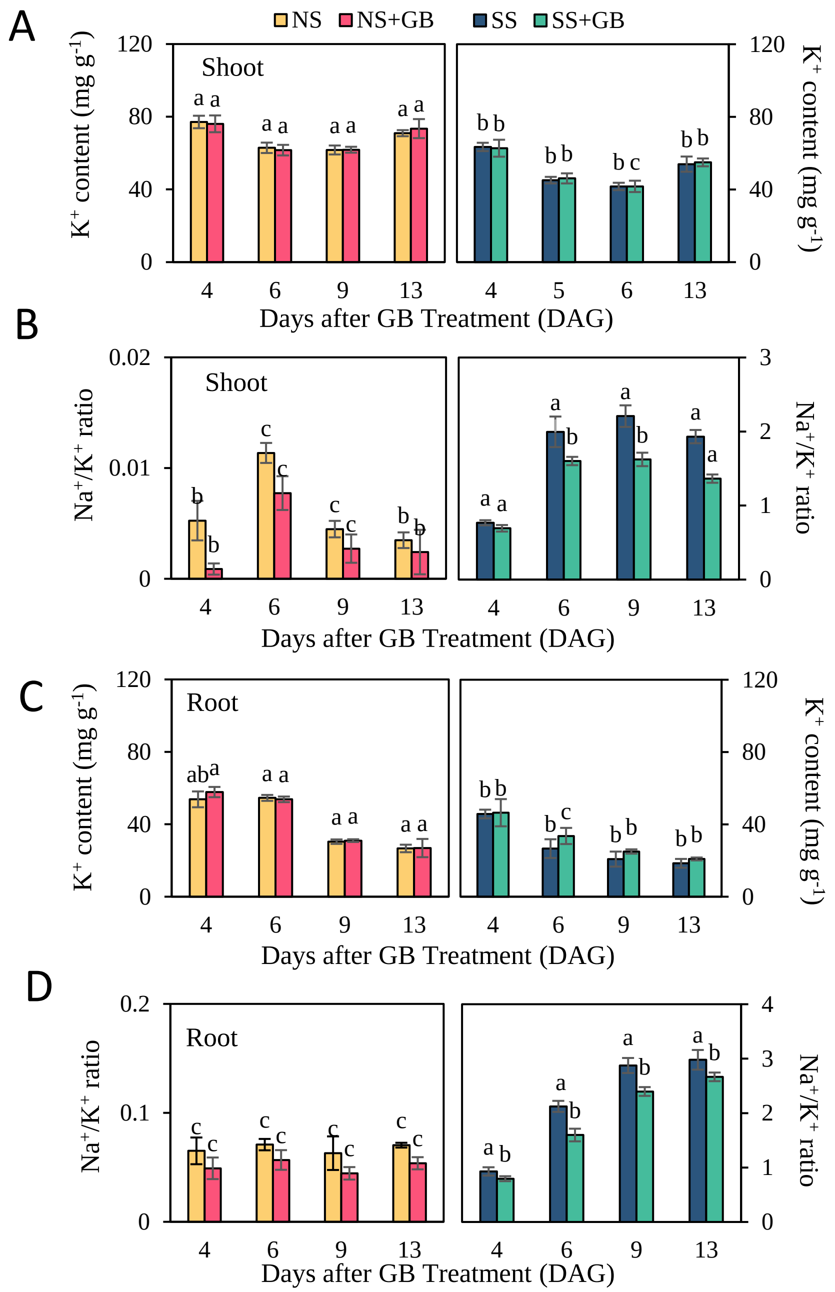

Supplement: Supplementary file 1 [file DataSheet_1.pdf]
